# Sinus computed tomography predicts clinical response to corticosteroids in chronic rhinosinusitis with nasal polyps

**DOI:** 10.1186/s13601-018-0211-1

**Published:** 2018-07-02

**Authors:** Haiyu Hong, Dan Wang, Kai Sen Tan, Rui Zheng, Fenghong Chen, Wenxiang Gao, Haixin He, Jianbo Shi, Yunping Fan, Qintai Yang, Yueqi Sun

**Affiliations:** 1 Allergy Center, Department of Otolaryngology, The Fifth Affiliated Hospital of Sun Yat-sen University, No. 52, Meihua East Road, Zhuhai, 519020 China; 2 Otorhinolaryngology Hospital, The First Affiliated Hospital, Sun Yat-sen University, 58 Zhongshan Road II, Guangzhou, 510080 Guangdong China; 3 Department of Otolaryngology, National University of Singapore, National University Health System, Singapore, Singapore; 4 Department of Otolaryngology, The Third Affiliated Hospital of Sun Yat-sen University, No. 600, Tianhe Road, Guangzhou, 510630 China

**Keywords:** Chronic rhinosinusitis, Computed tomography, Glucocorticoid therapy, Logistic regression

## Abstract

**Background:**

Chronic rhinosinusitis with nasal polyps (CRSwNP) is a heterogeneous inflammatory disease usually characterized by chronic eosinophilia in the sinonasal mucosa, which often requires glucocorticoid (GC) therapy. However, the therapeutic response varies markedly between individuals. The objective of this study was to evaluate the diagnostic values of sinus computed tomography (CT) for GC-sensitivity in patients with CRSwNP.

**Methods:**

We conducted a prospective, single-blinded study of 47 consecutive patients with CRSwNP. These patients were given a course of oral prednisone (30 mg daily for 14 days) and subsequently classified into objectively GC-sensitive and -insensitive subgroup according to the change in nasal polyp size score, or subjectively GC-sensitive and -insensitive subgroup according to the change in total nasal symptom score. The following parameters were compared between GC-sensitive and GC-insensitive subgroups: Lund-Mackay scores, olfactory cleft (OC) scores, and blood eosinophil counts and ratio (percentage of the total white blood cells).

**Results:**

25/47 (53.2%) and 29/47 (61.7%) patients were objectively and subjectively sensitive to GC therapy, respectively. The OC score and the blood eosinophil counts and ratio in GC-sensitive subgroup were significantly higher than those in GC-insensitive subgroup, defined either objectively or subjectively. Multivariate logistic regression revealed that OC score was independent risk factor for objective or subjective GC-sensitivity. The OC score exhibited comparable accuracy with the blood eosinophil ratio as predictor of objective and subjective GC-sensitivity (the OC score AUC = 0.775 and 0.829, respectively). A OC score of 3.5 could act as a reliable indicator for predicting the clinical response to GC therapy in CRSwNP.

**Conclusion:**

Our prospective findings validate the potential value of sinus CT scan in predicting GC-sensitivity in CRSwNP patients.

**Electronic supplementary material:**

The online version of this article (10.1186/s13601-018-0211-1) contains supplementary material, which is available to authorized users.

## Background

Chronic rhinosinusitis (CRS) is a common clinical syndrome associated with persistent mucosal inflammation in the nasal sinus, affecting 14% of adults in the United States and 8% in China [1, 2]. CRS is primarily diagnosed through observation of symptoms and clinical signs and supplemented with computed tomography (CT) and nasal endoscopy. Based on the presence of nasal polyps during these diagnosis, CRS can be further classified into two subtypes: chronic rhinosinusitis with nasal polyps (CRSwNP) and chronic rhinosinusitis without nasal polyps (CRSsNP). Among these CRS manifestations, CRSwNP is known to show a higher degree of disease severity and poorer response to medical and surgical therapy [1]. In the western world, CRSwNP has been characterized by a T helper (Th) 2-biased response and tissue eosinophilia [3–5]. In China however, CRSwNP patients are presented with a more distinct pathogenic phenotype that involves neutrophilic accumulations and mixed Th1/Th2/Th17 response [6, 7], hence suggesting a more heterogeneous nature of CRSwNP in China.

Glucocorticoids (GC) are anti-inflammatory drugs frequently employed in the treatment of CRS and other inflammatory airway diseases like asthma and chronic obstructive pulmonary disease (COPD). However, the therapeutic response varies markedly between individuals for these treatments. It has been reported that 5–10% of asthmatic patients display insensitivity to corticosteroids [8]; while the prevalence of GC insensitivity in CRSwNP is unclear. Nevertheless, it is still reasonable to deduce that there are a number of CRSwNP patients with GC insensitivity in China, as the additional neutrophilic infiltrate in nasal polyps may reduce the efficacy of oral GC treatment [6]. Therefore, GC insensitivity in CRSwNP may represent a problem in the management and treatment of the disease, where extra determinants to GC treatment responses using clinical parameters are highly warranted.

So far, studies on predicting the response to GC in CRS patients are limited. Though recently, Sinus CT scores was shown to have high predictive value for identifying eosinophilic CRSwNP [9]. Taking into account that eosinophilic airway inflammation is predisposed to be GC sensitive [10], we therefore hypothesized that sinus CT scores could be used to predict the clinical response of GC treatment in patients with CRSwNP. The aim of this study is to evaluate the sinus CT features between GC-sensitive and GC-insensitive CRSwNP patients to assess the diagnostic values of sinus CT scores in predicting GC-sensitivity in patients with CRSwNP.

## Methods

### Subjects and study design

The study design was a prospective, single-blinded and single-center study where forty-seven patients with CRSwNP were recruited from the Fifth Affiliated Hospital of Sun Yat-sen University (Zhuhai, China). This study was approved by the hospital ethics committee (Approved No. of ethic committee: 2015-S39), and written informed consent was obtained from each subject. The diagnosis of CRSwNP was carried out according to the European position paper on rhinosinusitis and nasal polyps (EPOS 2012) [1]. All patients showed a score of 1–4 on both sides based on the nasal polyp size score (NPSS) by nasal endoscopy (Additional file 1: Table S1) [11, 12]. The total nasal symptom score (TNSS) was calculated as the sum of 4 individual patient-assessed symptom scores for nasal congestion, loss of smell, anterior rhinorrhea, and postnasal drip, each evaluated using a scale of 0 = None, 1 = Mild, 2 = Moderate, or 3 = Severe [1, 6]. All TNSS and NPSS were evaluated and scored by an otolaryngologist who was blinded to the design of the trial. Prior to GC treatment, all patients were evaluated for CT scores and peripheral blood eosinophilia followed by a prescription of oral GC (30 mg of prednisone once daily for 14 days). Before and after oral GC therapy, NPSS and TNSS were recorded. In the run-in phrase of this study, we evaluated the GC responsiveness based on the TNSS subjectively and the NPSS objectively, and consequently found that the GC responsiveness objectively determined by NPSS and that subjectively determined by TNSS were both efficient in reflecting the alteration of eosinophilic inflammation in polyp tissues (data not shown). Therefore, in the present study, the GC responsiveness of all patients was determined based on the changes of TNSS and NPSS after a 2-week oral prednisone treatment compared with the baseline. The diagnosis of asthma was determined by a pneumologist. Diagnosis of aspirin intolerance and allergic rhinitis is based on detailed clinical history. Atopic status was evaluated by a skin prick test or the serum IgE (Phadia, Uppsala, Sweden) specific to common inhaled allergens (e.g. pollens, house dust mites, pets, molds, and cockroaches). None of the patients included used oral or nasal steroids or other immunomodulatory drugs within 4 weeks before starting oral GC.

### Computed tomography (CT) imaging and Sinus Score

Each participant underwent sinus CT scanning in a supine position. 1-mm axial images were collected and reconstructed offline and reformatted to 3-mm coronal images for analysis (Toshiba Aquilion, Tokyo, Japan). Sinus CT images were reviewed by an investigator blinded to the patients’ clinical condition. CT scoring was based on the Lund-Mackay scoring system [13], supplemented with the olfactory cleft (OC) score. In the Lund-Mackay staging system, which is based on a simple numeric score derived from the CT images, each sinus group is assigned a numeric grade: 0, no abnormality; 1, partial opacification; and 2, total opacification. The following scores were also determined: maxillary sinus score (M score), anterior ethmoid sinus score (AE score), posterior ethmoid sinus score (PE score), sphenoid sinus score (S score), frontal sinus score (F score) and the ostiomeatal complex score (OMC score). The OC score was observed on the posterior ethmoid sinus and superior turbinate level [14]. Each OC was graded in a similar manner to that used in the Lund–Mackay staging system: 0, no opacification; 1, partial opacification; and 2, total opacification. Three additional scores were calculated from the Lund–Mackay scores: total ethmoid sinus score (E score; sum of the AE and PE scores), E/M ratio (ratio of the E and M scores) and PE/AE ratio (ratio of the PE and AE scores).

### Statistical analysis

IBM SPSS statistical software, version 20.0 (IBM, Inc., Chicago, Illinois), was used for statistical analysis. Continuous variables were given as mean ± SD, and categorical variables were given as number (percentage) of the total population. The unpaired t test was used to compare mean values of the items with standard normal distributions between GC-sensitive and GC-insensitive subgroup. Categorical variables were analyzed with the χ^2^ test. Because values of items were not normally distributed, the Mann–Whitney test for unpaired comparisons was used. Binary logistic regression analysis was performed for multivariate analysis of predictive factors of GC-sensitivity and odds ratio calculation. The receiver operating characteristic curve analyzed the predict ability of OC scores and other clinical parameters. *P* < .05 was considered statistically significant.

## Results

The subjects were classified as objectively GC-sensitive subgroup and objectively GC-insensitive subgroup after completion of GC prescription, based on the endoscopic score criterion of Milara et al. [11, 12], we designated patients who are unable to reduce more than 1 point in the nasal polyp scoring system after oral corticosteroid management as GC-insensitive CRSwNP. Meanwhile, to evaluate the subjective response to oral corticosteroids, we divided the subjects into subjectively GC-sensitive CRSwNP and subjectively GC-insensitive CRSwNP based on a similar system, namely patients who are unable to reduce more than 1 point in the TNSS system after oral corticosteroid management were classified as subjectively GC-insensitive CRSwNP.

As a result, there are 25 patients (53.2%) in the objectively GC-sensitive subgroup and 22 patients (46.8%) in the objectively GC-insensitive subgroup. When subjects were classified subjectively, there are 29 patients (61.7%) in the subjectively GC-sensitive subgroup and 18 patients (38.3%) in the subjectively GC-insensitive subgroup. The demographic and clinical characteristics of the subgroups were showed in Table 1. As expected, we found the mean values of the blood eosinophil counts and ratio, as well as the proportion of patients with concurrent asthma were significantly higher in objectively GC-sensitive subgroup and subjectively GC-sensitive subgroup than those in objectively GC-insensitive subgroup and subjectively GC-insensitive subgroup, respectively (all *P* < .05). The mean values of NPSS and TNSS after GC therapy were significantly higher in objectively and subjectively GC-insensitive subgroups than those in objectively and subjectively GC-sensitive subgroups, respectively (all *P* < .05). Interestingly, we also found the mean value of TNSS before GC therapy was significantly higher in subjectively GC-sensitive subgroup than that in subjectively GC-insensitive subgroup (*P* = .01). The mean age of subjectively GC-sensitive subgroup was significantly higher than that of subjectively GC-insensitive subgroup (*P* = .03).

**Table 1:** Demographic data and blood eosinophil counts of patients with GC-sensitive NP and GC-insensitive NP

	Objective sensitivity			Subjective sensitivity		
	GC-sensitive (n = 25)	GC-insensitive (n = 22)	*P* value	GC-sensitive (n = 29)	GC-insensitive (n = 18)	*P* value
Male, NO. (%)	16 (64)	12 (54.5)	.56	18 (62)	10 (55.6)	.76
Age, mean (SD) (years)	39.5 (12.7)	34.8 (11.9)	.21	40.4 (11.7)	32.4 (12.3)	.03
Disease duration (years)	6.4 (8.4)	8.5 (9.0)	.4	7.8 (10.1)	6.8 (6.0)	.7
Smoking, NO. (%)	3 (12)	3 (13.6)	> .99	5 (17.2)	1 (5.6)	.38
Eosinophil count, mean (SD) (cells/μL)	404.4 (200.3)	209.5 (233.1)	.003	401.4 (213.6)	171.1 (200.2)	< .001
Eosinophil ratio, mean (SD)	5.25 (2.59)	3.17 (3.46)	.02	5.39 (2.76)	2.48 (2.99)	< .001
NPSS before treatment, mean (SD)	5.72 (1.37)	5.64 (1.29)	.83	5.76 (1.38)	5.56 (1.25)	.61
NPSS after treatment, mean (SD)	4.28 (1.46)	5.77 (1.31)	< .001	4.55 (1.62)	5.67 (1.24)	.02
TNSS before treatment, mean (SD)	8.8 (2.25)	8.05 (1.86)	.22	9.07 (1.71)	7.44 (2.31)	.01
TNSS after treatment, mean (SD)	6.5 (1.71)	7.59 (1.79)	.04	6.53 (1.68)	7.78 (1.8)	.02
Atopy, NO. (%)	17 (68)	13 (59.1)	.45	21 (72.4)	9 (50)	.16
Asthma, NO. (%)	11 (44)	3 (13.6)	.02	12 (41.4)	2 (11.1)	.03
AR, NO. (%)	11 (44)	7 (31.8)	.4	14 (48.3)	4 (22.2)	.12
AERD, NO. (%)	1 (4)	1 (4.5)	.93	1 (3.4)	1 (5.6)	.73

GC, glucocorticoid; NPSS, nasal polyp size score; TNSS, total nasal symptom score; AR, allergic rhinitis; AERD, aspirin-exacerbated respiratory disease

For sinus Lund-Mackay scoring, the OC score in GC-sensitive subgroup defined either objectively or subjectively was significantly higher than that in GC-insensitive subgroup (both *P* < 0.001). The PE score, E score and PE/AE score were significantly higher in subjectively GC-sensitive subgroup than that in subjectively GC-insensitive subgroup (*P* < .001, = .01, and = .02, respectively). Similar trends were observed in objectively GC-sensitive subgroup, although they were not statistically significant (*P* = .06, .08 and .09, respectively). (Table 2) Multivariate logistic regression analysis revealed that only the OC score was significantly different between GC-sensitive and GC-insensitive subgroup defined either objectively or subjectively (*P* = .009 and .01, respectively) (Table 3).

**Table 2:** Sinus computed tomography scores for patients with GC-sensitive NP and GC-insensitive NP

	Objective sensitivity			Subjective sensitivity		
	GC-sensitive (n = 25)	GC-insensitive (n = 22)	*P* value	GC-sensitive (n = 29)	GC-insensitive (n = 18)	*P* value
M score	2.9 (0.9)	3.0 (1.0)	.54	2.8 (0.9)	3.2 (1.0)	.23
AE score	3.7 (0.7)	3.5 (0.9)	.22	3.7 (0.7)	3.4 (0.9)	.24
PE score	3.4 (1.2)	2.6 (1.6)	.06	3.4 (1.2)	2.3 (1.6)	< .001
E score	7.1 (1.6)	6.0 (2.2)	.08	7.1 (1.7)	5.7 (2.1)	.01
S score	2.7 (1.3)	2.5 (1.7)	.85	2.7 (1.4)	2.4 (1.7)	.84
F score	3.1 (1.4)	2.9 (1.7)	.8	3.1 (1.4)	2.9 (1.7)	.93
OMC score	3.6 (1.0)	3.5 (1.0)	.54	3.6 (1.0)	3.6 (0.96)	.73
OC score	3.6 (1.0)	2.2 (1.4)	< .001	3.6 (1.0)	1.9 (1.3)	< .001
E/M ratio	2.5 (0.8)	2.3 (1.2)	.18	2.5 (0.9)	2.2 (1.2)	.12
PE/AE ratio	0.9 (0.4)	0.7 (0.4)	.09	0.9 (0.3)	0.7 (0.4)	.02

GC, glucocorticoid; M score, maxillary sinus score; AE score, anterior ethmoid sinus score; PE, posterior ethmoid sinus score; E score, total ethmoid sinus score; S score, sphenoid sinus score; F score, frontal sinus score; OMC score, ostiomeatal complex score; OC score, olfactory cleft score; E/M ratio, ratio of the E and M scores; PE/AE ratio, ratio of the PE and AE scores

**Table 3:** Multivariate logistic regression analysis of the predictive factors for GC sensitivity

	Objective sensitivity		Subjective sensitivity	
	OR (95% CI)	*P* value	OR (95% CI)	*P* value
PE score	0.093 (0.001–7.174)	.284	16.287 (0.18–1477.535)	.225
E score	2.903 (0.369–22.815)	.311	0.265 (0.028–2.489)	.245
OC score	2.882 (1.301–6.384)	.009	2.508 (1.248–5.039)	.01
PE/AE ratio	38.067 (0.024–59,285.002)	.332	0.032 (0–20.094)	.749

E score, total ethmoid sinus score; OC score, olfactory cleft score; PE/AE ratio, ratio of the PE and AE scores; CI, confidence interval; OR, odds ratio

**Fig. 1:**
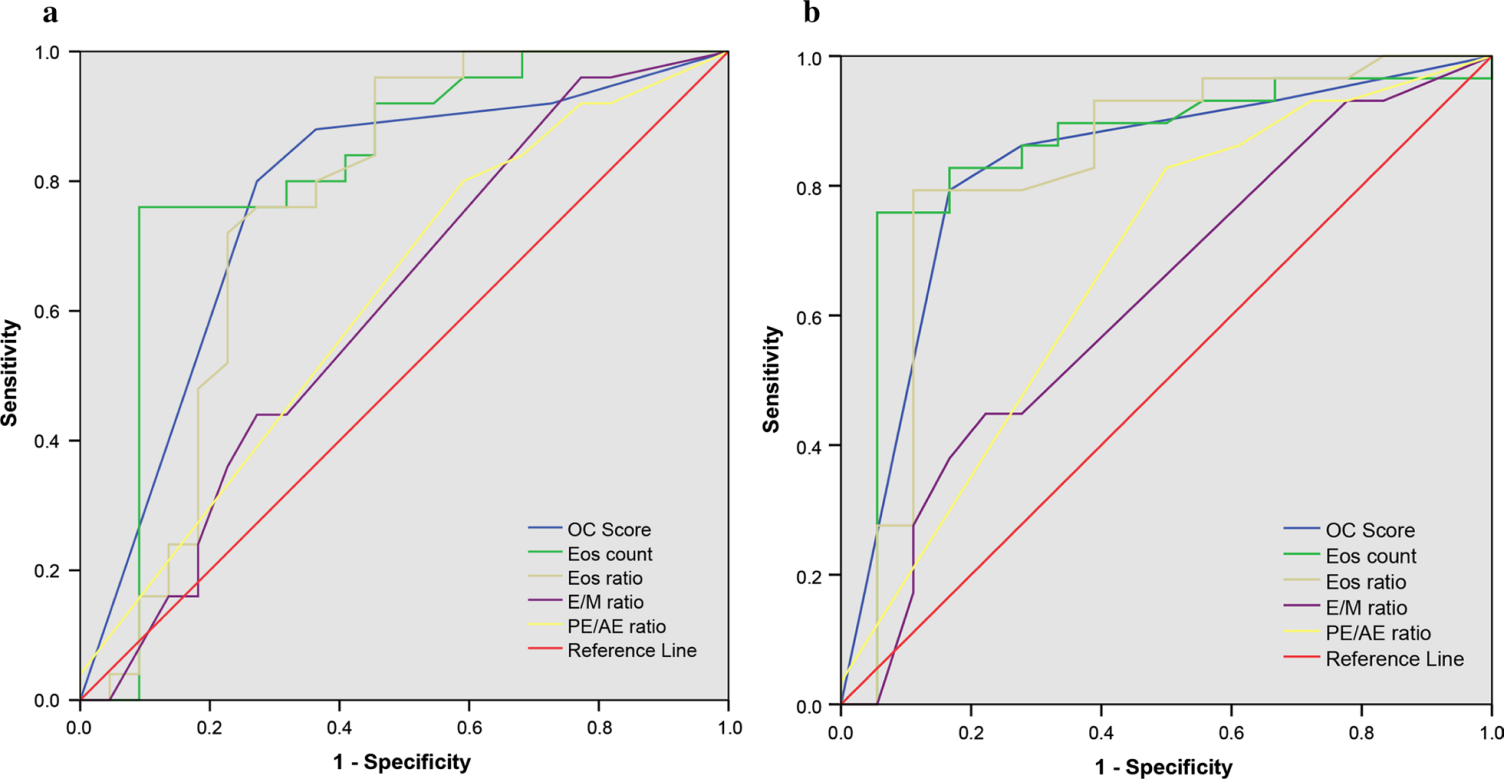
The receiver operating characteristic (ROC) curve for olfactory cleft (OC) score and other clinical parameters. Area under the ROC curve (AUC) of OC score was 0.775 (95% confidence interval, 0.633–0.916) for objective GC-sensitivity (**a**), and 0.829 (95% confidence interval, 0.699–0.958) for subjective GC-sensitivity (**b**)

Figure 1 showed the receiver operating characteristic curves (ROC) for the OC scores, eosinophil counts and ratio, as well as E/M ratio and PE/AE ratio. The area under the curve (AUC) values for each clinical parameter are shown in Table 4. As expected, the eosinophil counts had the highest AUC value (0.815 and 0.85 for objective and subjective GC-sensitivity, respectively). Surprisingly, the OC score exhibited comparable accuracy with the blood eosinophil ratio as predictor of objective and subjective GC-sensitivity (the OC score AUC = 0.775 and 0.829, respectively). Three cutoff points for the OC score were selected and the sensitivity and specificity were calculated (Table 5). When the OC score was 3.5 or higher, the sensitivity was 80.0 and 79.3%, specificity was 72.7 and 83.3%, for predicting objective and subjective GC-sensitivity, respectively.

**Table 4:** AUCs for individual clinical markers

	Objective sensitivity			Subjective sensitivity		
	AUC	OR (95% CI)	*P* value	AUC	OR (95% CI)	*P* value
Eosinophil count	0.815	0.68–0.951	< .001	0.85	0.721–0.978	< .001
Eosinophil ratio	0.76	0.61–0.91	.002	0.829	0.692–0.966	< .001
OC score	0.775	0.633–0.916	.001	0.829	0.699–0.958	< .001
E/M ratio	0.606	0.441–0.772	.212	0.628	0.461–0.795	.143
PE/AE ratio	0.619	0.457–0.781	.163	0.675	0.512–0.839	.045

AUC, area under the receiver operator characteristic curve; CI, confidence interval; OR, odds ratio; OC score, olfactory cleft score; E/M ratio, ratio of the total ethmoid sinus scores and maxillary sinus scores; PE/AE ratio, ratio of the posterior ethmoid sinus scores and anterior ethmoid sinus scores

**Table 5:** Sensitivity and specificity of OC score for predicting GC-sensitivity at different thresholds

	Cut-off point	Objective sensitivity		Subjective sensitivity	
		Sensitivity (%)	Specificity (%)	Sensitivity (%)	Specificity (%)
OC score	≧ 1.5	0.92	0.273	0.931	0.333
	≧ 2.5	0.88	0.636	0.862	0.722
	≧ 3.5	0.8	0.727	0.793	0.833

OC score, olfactory cleft score

## Discussion

CRSwNP with different pathological endotypes could display significant differences in their responses to GC treatment. Therefore, identification of additional clinical parameters that could supplement the current understanding of GC clinical response prediction in patients with CRSwNP would greatly improve the management of the disease. In the present study, we found that the OC score could be used as a supplementary predictor of objective and subjective GC-sensitivity in CRSwNP patients. At an OC score of 3.5 or higher for predicting objective and subjective GC-sensitivity, the sensitivity reached 80.0 and 79.3%, the specificity was 72.7 and 83.3%, respectively. Moreover, we found the accuracy of OC score in predicting GC-sensitivity was comparable with the blood eosinophil ratio. To our knowledge, our study provided evidence that OC area opacification in sinus CT could be a main feature of GC-sensitive CRSwNP, and may help improve prediction of GC-sensitivity in CRSwNP management.

Although most GC resistance studies have focused on asthma, evidence is emerging that similar GC resistance is observed in CRS [15–18]. However, it is still unclear how prevalent the GC insensitivity is in CRSwNP. In this study, based on the changes from baseline in objective and subjective clinical parameters (NPSS and TNSS) in patients treated with oral prednisone, we showed 46.8% of CRSwNP patients was objectively GC-insensitive, and 38.3% was subjectively GC-insensitive. Nevertheless, it should be noted that GC-sensitivity is not an absolute but relative concept, thus the prevalence of GC-sensitivity in CRS can be varied depending on how it is defined. In the present study, we used the NPSS to determine the GC-sensitivity according to the studies reported by Milara et al. [11, 12]. Their findings suggest that the GC-sensitivity determined by the change in NPSS system after GC treatment could reflect the pathogenesis of GC resistance in CRSwNP. In addition, in the run-in phrase of the present study, we found GC-sensitivity determined by NPSS and TNSS are both efficient in reflecting the alteration of eosinophilic inflammation in polyp tissues (Data not shown). Therefore, the concept of GC-sensitivity in the present study was defined based on the changes of TNSS and NPSS after a 2-week oral prednisone treatment compared with the baseline.

Previous studies have shown that increased expressions of pro-inflammatory cytokines, such as interleukin-1β, intercellular adhesion molecule 1, nuclear factor-κB, glucocorticoid receptor-β, transforming growth factor-β1 and mucin 4, in nasal polyp tissues were associated with a poor response to GC treatment, suggesting these pro-inflammatory cytokines might be involved in pathogenesis of GC resistance and could potentially be as biomarkers of GC insensitivity in CRSwNP patients [11, 19, 20]. In terms of clinical parameters, we have reported that smell loss score, ethmoid osteitis index and blood eosinophil number and ratio could be used as the surrogate markers for the diagnosis of eosinophilic CRS [21]. Recently, Meng et al. [9] found that the ratio of the CT scores for the ethmoid sinus and maxillary sinus (E/M ratio) had superior predictive value over other clinical parameters including blood eosinophil counts. Although the association between eosinophilic mucosal inflammation and high GC-sensitivity has been well established, it would be an interesting addition to the predictive scoring system if we can also employ atraumatic clinical parameters to predict the clinical responses of GC therapy. Therefore, based on this study, we showed that the OC score had a high diagnostic accuracy in identifying both objectively and subjectively GC-sensitive patients with CRSwNP, adding the clinical application value of CT for the diagnosis of CRSwNP as well as supplementing the current prediction parameters.

Increased eosinophil counts in the sputum and peripheral blood have been well recognized as biomarkers of GC-sensitive patients with asthma [22, 23]. In the present study, we also showed that both blood eosinophil counts and ratio had high predictive values for diagnosing GC-sensitivity in CRSwNP patients, suggesting that peripheral blood eosinophil number could be alternatively used for the prediction of GC-sensitivity in CRSwNP patients when CT scanning is unavailable; or together to improve the sensitivity and specificity of predicting GC-sensitivity.

In this study, we found that the baseline TNSS in subjectively GC-sensitive subgroup was significantly higher than that in subjectively GC-insensitive subgroup. It is likely because that the subjectively GC-sensitive CRSwNP had significantly higher peripheral blood eosinophil counts, suggesting a higher constitution of eosinophilic CRSwNP [24], which is likely to have worse symptoms [25, 26].

Our current study is limited because the baseline NPSS of CRSwNP patients recruited in our study were mostly more than 4, restricting the ability to extrapolate our findings to the general CRSwNP patients. Moreover, this study was done with a small sample size and lack of a long-term GC therapy group. Therefore, the results of the present study need to be validated in a larger independent study to assess their variability and validity.

## Conclusions

Our study provided primary evidence that bilateral OC opacification in CT is a specific feature in GC sensitive CRSwNP patient. OC score could be a useful addition in the parameters analysed for the prediction of sensitivity in patients with CRSwNP.

## Additional file

**Additional file 1: Table S1.** Nasal polyp size score.

## Abbreviations

AE score: anterior ethmoid sinus score
AUC: area under the curve
COPD: chronic obstructive pulmonary disease
CT: computed tomography
CRS: chronic rhinosinusitis
CRSsNP: chronic rhinosinusitis without nasal polyps
CRSwNP: chronic rhinosinusitis with nasal polyps
E score: total ethmoid sinus score
EPOS: European position paper on rhinosinusitis and nasal polyps
F score: frontal sinus score
GC: glucocorticoid
M score: maxillary sinus score
NPSS: nasal polyp size score
OC: olfactory cleft
OMC score: ostiomeatal complex score
PE score: posterior ethmoid sinus score
ROC: receiver operating characteristic curves
S score: sphenoid sinus score
SD: standard deviation
Th: T helper
TNSS: total nasal symptom score
