# Supplementary material for: Sinus computed tomography predicts clinical response to corticosteroids in chronic rhinosinusitis with nasal polyps
Source: Clin Transl Allergy. 2018 Jul 2;8:24. doi: 10.1186/s13601-018-0211-1 (PMC6027579; doi:10.1186/s13601-018-0211-1)
Supplement: Supplementary file 1 — Additional file 1: Table S1. Nasal polyp size score. [file 13601_2018_211_MOESM1_ESM.docx]

**Table S1** Nasal polyp size score

| **Polyp score** | **Polyp size** |
| --- | --- |
| 0 | No polyps |
| 1 | Small polyps in the middle meatus not reaching below the inferior border of the middle concha. |
| 2 | Polyps reaching below the lower border of the middle turbinate. |
| 3 | Large polyps reaching the lower border of the attachment of inferior turbinate or polyps medial to the middle concha. |
| 4 | Large polyps causing almost complete congestion/obstruction of the inferior meatus. |
